# Neuroangiostrongyliasis: Global Spread of an Emerging Tropical Disease

**DOI:** 10.4269/ajtmh.22-0360

**Published:** 2022-11-07

**Authors:** Robert H. Cowie, Vernon Ansdell, Claire Panosian Dunavan, Randi L. Rollins

**Affiliations:** ^1^ Pacific Biosciences Research Center, University of Hawaii, Honolulu, Hawaii;; ^2^ John A. Burns School of Medicine, University of Hawaii, Honolulu, Hawaii;; ^3^ David Geffen School of Medicine, University of California, Los Angeles, California;; ^4^ School of Life Sciences, University of Hawaii, Honolulu, Hawaii

## Abstract

Neuroangiostrongyliasis (NAS) is an emerging parasitic disease caused by the neurotropic nematode *Angiostrongylus cantonensis*. Since it was first discovered, in rats in southern China in the 1930s, this tropical to subtropical parasite has spread to much of Southeast Asia, the Pacific Islands (including Hawaii), Australia, Japan, South America, the southeastern United States, the Caribbean, Africa, the Canary Islands, and the Balearic Islands. The parasite completes its natural life cycle in snails and slugs (intermediate hosts), and rats (definitive hosts). Humans become accidental hosts after ingesting infective third-stage larvae contained within uncooked or undercooked intermediate or paratenic hosts, an event that sometimes results in NAS, also known as rat lungworm disease. Although *A. cantonensis* larvae cannot complete their life cycle in humans, their migration into the brain and spinal cord combined with a powerful inflammatory reaction often leads to eosinophilic meningitis and can, in rare instances, lead to coma, paralysis, and death or, in other cases, chronic, disabling neurologic sequelae. Symptoms of NAS are diverse, which often makes it difficult to diagnose. Treatment may include administration of analgesics, corticosteroids, anthelminthics, and repeat lumbar punctures to reduce intracranial pressure. Unfortunately, few medical providers, even in endemic areas, are familiar with *A. cantonensis* or its epidemiology, diagnosis, and treatment. As the parasite continues to spread and NAS affects more people, medical practitioners, as well as the general public, must become more aware of this emerging zoonosis and the potentially devastating harm it can cause.

## INTRODUCTION

**Figure 1.**
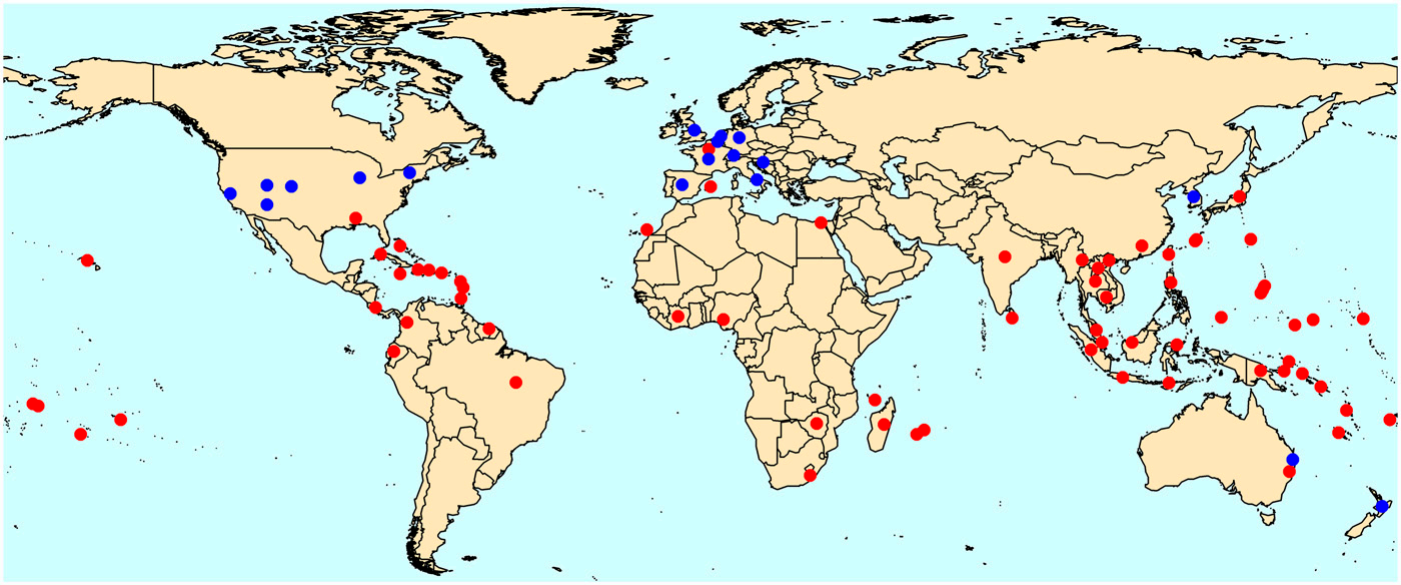
The global distribution, by country, of combined reported presence of *Angiostrongylus cantonensis* and cases of human or animal neuroangiostrongyliasis (red circles), with cases in returning travelers to nonendemic areas distinguished (blue circles).^2^^,^^4^^–^^19^ If part of a country is distant from the main part of the country—Guam, Saipan, Hawaii, American Samoa, and Puerto Rico (United States); Ryukyu and Ogasawara Islands (Japan); New Caledonia, Tahiti, Mayotte, Réunion, Guadeloupe, and Martinique (France); and Canary Islands and Mallorca (Spain)—dots are placed on those locations in addition to the main part of country (if also present there). For most countries/territories, a single dot has been placed roughly where the greatest concentration of records occurs. For countries with records on multiple widespread islands, dots are placed on those areas with records—Malaysia (Peninsula Malaysia, Sarawak), Indonesia (Java, Sumatra, Flores, Sulawesi), and the Federated States of Micronesia (Chuuk, Pohnpei). For the continental United States, several dots for returning travelers are placed roughly where they were diagnosed. Note that the cases in the Bahamas may have originated in Louisiana, and the presence of *A. cantonensis* in Zimbabwe is unconfirmed.

*Angiostrongylus cantonensis* was first discovered in rats in southern China in 1933; this area is generally accepted as the parasite’s region of origin. The first known human case of neuroangiostrongyliasis (NAS) was identified in nearby Taiwan in 1944, although appreciated only after the parasite and the disease were clearly linked almost two decades later.^1^ From this region, the parasite spread westward through Southeast Asia, eastward to islands of the Pacific, north to Japan, and south to Australia, no doubt associated with military movements during and immediately after World War II, and increasing travel and trade during the latter half of the 20th century.^2^ *Angiostrongylus cantonensis* was then found in a number of Caribbean islands and in the southeastern United States, where it was first reported in 1988,^3^ and more recently in South America^4^^,^^5^ (Figure 1). Reports of NAS often precede detection of *A. cantonensis* in regional faunas, as likely animal hosts are rarely screened until a human case arises. Nonetheless, *A. cantonensis* has most recently been found in rodents in the Canary Islands^19^ and hedgehogs in the Balearic Islands of the Mediterranean,^14^ where NAS has not yet been reported. Human cases are also rising in travelers returning from endemic regions,^12^^,^^16^^,^^17^ and there has even been an enigmatic case in northern France that did not involve travel or known ingestion of imported food.^16^ This review takes a global perspective, while focusing in somewhat greater detail on the United States, including Hawaii.

## INFECTION PATHWAYS

**Figure 2.**
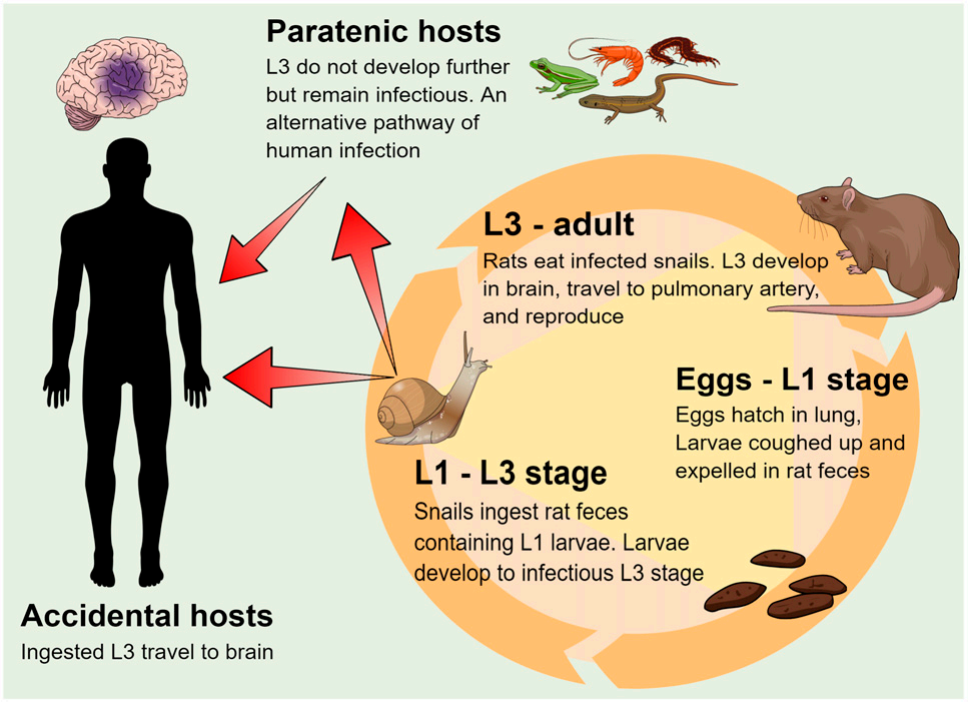
*Angiostrongylus cantonensis* completes its life cycle in various species of rats (definitive hosts) and snails (intermediate hosts).^9^^,^^20^^,^^21^ Snails become infected by ingesting rat feces containing freshly hatched larvae (L1). The larvae develop into infectious third-stage larvae (L3) in the snail and remain as such for the life span of the snail or until the snail is eaten by a definitive, paratenic, or accidental host.

To understand the parasite’s transmission to humans, one must understand its life cycle (Figure 2). Rats (definitive hosts) and snails and slugs (intermediate hosts; hereafter “snails”) are both required to complete the natural life cycle.^9^ Paratenic hosts (e.g., freshwater prawns, frogs and toads, and land crabs, with centipedes most recently reported),^22^ in which the infectious stage 3 larvae (L3) are acquired by ingestion of intermediate hosts or other paratenic hosts carrying L3, do not support development of the L3. However, paratenic hosts, in which the L3 remain dormant, can still infect accidental hosts (Figure 2). Humans, and various other animals, are accidental hosts in which the infectious L3 can neither develop beyond the subadult stage (L5) nor reproduce.^9^ Instead, after accidental hosts acquire L3 by ingesting infected intermediate or paratenic hosts, their L3 quickly reach the central nervous system, primarily the brain, where they feed, grow, molt, and eventually die, as opposed to returning to the pulmonary artery to reproduce, as would normally occur in a rat (Figure 2).

Raw or poorly cooked intermediate or paratenic hosts carrying L3 are the classic vehicles of human infection. Raw snails are sometimes eaten on a dare or for a bet, as reported in Louisiana,^23^ Hawaii,^24^^,^^25^ Okinawa,^26^ New Caledonia,^27^ Australia,^28^^,^^29^ and Brazil (S. Thiengo, personal communication). Raw or undercooked snails are also eaten as delicacies, especially in parts of China and Thailand.^30^^,^^31^ Among paratenic hosts,^22^ freshwater prawns are widely eaten raw, especially in French Polynesia, where they have been heavily implicated in epidemics of NAS since the 1950s.^32^ The liver, meat, tongue, and testes of monitor lizards are eaten in India, Sri Lanka, Thailand, and Laos, primarily by men who believe these organs will improve their strength and virility.^33^^–^^35^ Other paratenic hosts eaten raw include land crabs and centipedes,^32^^,^^36^ as well as frogs and toads sometimes consumed for health purposes,^37^ and in one case in Louisiana on a dare.^38^ Obtaining a history of eating one of these intermediate or paratenic hosts raw can facilitate an early diagnosis of NAS.

However, in the absence of other clues, inadvertent ingestion of intermediate hosts (snails)—in particular, a whole or partial snail hidden in produce, especially leafy greens—is often the likeliest pathway of infection. In recent years, raw, blended vegetable juice (including so-called “green smoothies”) contaminated with infective larvae from snails accidentally blended along with the vegetables has been identified as another important source of infection.^39^ Only in rare cases, however, is there good circumstantial evidence for this; for example, among a group of students returning to the United States from a vacation in Jamaica, those who had eaten a Caesar salad on their last evening became ill with NAS, whereas those who had not done so remained uninfected and healthy.^40^ Even more rarely is there definitive proof. Nonetheless, inadvertent ingestion of intermediate hosts is probably the most important means of infection in many regions, including Hawaii. Paratenic hosts—namely, flatworms^41^^,^^42^—may also contaminate fresh leafy greens and other produce.

Additional infection pathways have been suggested.^32^ For example, can one become infected with *A. cantonensis* L3 by ingesting the slime trail laid down by an infected snail on a lettuce leaf? Several studies addressing this question have in fact demonstrated little or no release of L3 in slime.^43^ In Hawaii, some researchers have speculated that snails that drown in rainfall catchment tanks may release sufficient numbers of L3 to cause NAS in individuals who drink the contaminated water.^44^ Although large numbers of L3 have been observed leaving dead and dying snails (R. L. Rollins and R. H. Cowie, unpublished data), there are no data on the prevalence or density of *A. cantonensis* L3 in catchment water.

It is commonly assumed that the severity of disease in a patient with NAS reflects the number of L3 that were ingested. In this regard, it bears remembering that even very small snails can carry high numbers of L3, sometimes in the thousands.

## SIGNS AND SYMPTOMS, DIAGNOSIS AND TREATMENT

Infection with *A. cantonensis* is one of the leading causes of eosinophilic meningitis worldwide. After humans become infected, the most dramatic clinical findings reflect the presence of larvae in neurological tissues combined with a strong inflammatory reaction, especially as the larvae die. Occasionally, subadult worms as long as 1 to 3 cm appear in the eye, requiring surgical removal.^45^

Signs and symptoms are diverse, varying from patient to patient over days to weeks postinfection.^46^ There may be a prodromal phase as neurotropic L3 migrate from the intestinal tract, producing symptoms such as abdominal pain, nausea, vomiting, cough, dyspnea, headache, and a low-grade fever reflecting inflammation at multiple sites where the larvae lodge.^47^ When sought actively, early manifestations such as a rash (with or without pruritus), myalgias, and arthralgias probably occur in more than 20% of patients,^48^^,^^49^ sometimes within hours to days after infection (C. Panosian Dunavan, unpublished data).^50^^,^^51^ Although such symptoms are often overlooked or underreported, when sought actively and recognized, they can provide valuable clues enabling early presumptive diagnosis at a time when treatment with anthelminthics such as albendazole is particularly effective.^47^^,^^52^^,^^53^

Clinical features tend to be more specific after the parasites reach the central nervous system. Patients may experience migratory paresthesias and hyperesthesias in different parts of the body accompanied by myalgias often involving the neck and shoulders; severe, unremitting headaches; and bowel and bladder dysfunction resulting from radiculomyelitis.^8^ Occasionally, hydrocephalus, encephalitis, and cranial nerve palsies—even coma or death—later ensue. Some patients may suffer long-term disabilities with life-changing consequences; unfortunately, this form of the disease has been underappreciated for many years, especially in regions such as Australia and Hawaii, where the numbers of heavy infections seem to be rising.^54^^,^^55^

When NAS is suspected, a key diagnostic step is to perform a lumbar puncture to detect eosinophilia in the cerebrospinal fluid (CSF), and—in very rare cases—worms in the CSF, which confirms the diagnosis.^46^ However, because the signs and symptoms of NAS are diverse, delays in considering the diagnosis and performing lumbar punctures are common. This is true in both nonendemic and endemic regions, where few medical providers may be familiar with NAS. A valuable adjunct to diagnosis is the detection of *A. cantonensis* DNA in CSF via real-time polymerase chain reaction (PCR).^56^ More recent research has resulted in highly sensitive and ultrasensitive PCR tests that aim to detect very small amounts of *A. cantonensis* DNA not just in spinal fluid, but also in blood,^57^^,^^58^ possibly alleviating the need for lumbar puncture for diagnosis, although obtaining spinal fluid to exclude other diagnoses may still be necessary.

In some international laboratories, serological tests of blood and CSF have been used to detect acute infections resulting from *A. cantonensis*,^59^^,^^60^ but such tests are not available in the United States. In addition, because antibodies to *A. cantonensis* may take several weeks to develop after exposure to infective larvae, today, performing a DNA-based test is preferable in order to initiate treatment as early as possible. Last, in certain patients, empiric therapy may be justified based on epidemiological history and supportive clinical and laboratory findings including peripheral eosinophilia.

Following a diagnosis of NAS, many patients require analgesics for pain relief, and corticosteroids to lessen inflammation, sometimes together with repeat lumbar punctures to reduce intracranial pressure and relieve severe headaches. Additional options for pain relief include ketamine and intravenous lidocaine drugs.^50^^,^^61^^,^^62^ In challenging cases, a multidisciplinary approach to pain management is often necessary.

A final important therapy—anthelminthics to kill migrating worms—has been somewhat controversial, as some authors have speculated for decades that rapidly killing all the worms might produce a more damaging inflammatory reaction than would occur if subadult larvae died over a longer period of time.^8^^,^^63^ Nonetheless, a consensus has recently emerged that anthelminthics are useful, perhaps even key, especially if administered before or soon after the L3 reach the central nervous system, or at least before they molt and grow.^46^^,^^47^^,^^64^ It has also been suggested that anthelminthics be used prophylactically if, in an endemic area, a person realizes they may have been exposed to *A. cantonensis*, for instance by biting into a slug hidden in a sandwich; however, no study validating this approach is available.^47^

In short, avoiding the serious, sometimes devastating long-term consequences of severe infection should be a key objective for clinicians caring for patients with possible NAS. Early treatment with albendazole and corticosteroids, ideally taken within 2 weeks of infection, has been proposed as a way to limit chronic sequelae of the disease, despite the difficulty of diagnosing severely affected patients at such an early stage.^47^ Importantly, in a few cases, specific DNA in the CSF has been detected as early as 11 days after infection (V. Ansdell, unpublished data), and it would probably be detected even earlier in the CSF and blood using the previously mentioned highly sensitive or ultrasensitive PCR tests.^57^^,^^58^

## GLOBAL CASES OF NEUROANGIOSTRONGYLIASIS

*Angiostrongylus cantonensis* is generally considered a tropical and subtropical parasite, limited by low temperatures. The territories with the highest numbers of reported cases of NAS are by far Thailand and China, where eating raw or undercooked snails is the primary route of human infection, with French Polynesia, where raw prawns (paratenic hosts) are the leading source of infection, a distant third.^8^ The United States is next with the vast majority of human infections contracted in Hawaii (see below), followed by Cuba, New Caledonia, and Japan.^8^ In Australia there were 28 known cases between 1971 and 2018, of which at least seven were travelers who had recently returned from Vanuatu and Fiji, where the disease is endemic; five of the 28 died.^47^ Elsewhere, very few human cases have been reported.

**Figure 3.**
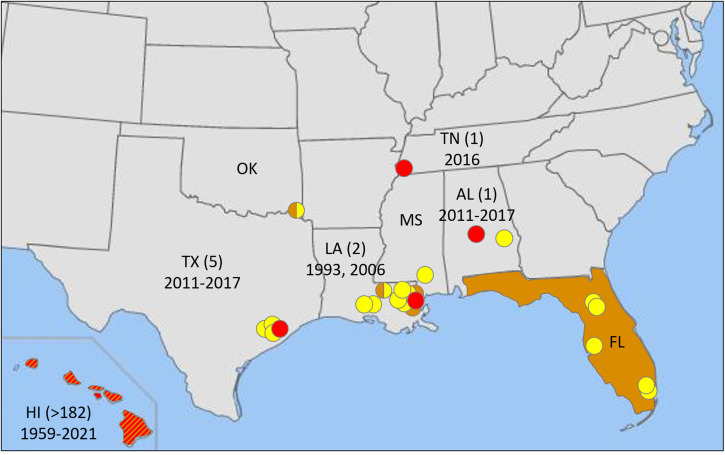
Known distribution of natural infections of hosts of *Angiostrongylus cantonensis*^65^^–^^67^ and autochthonous human cases of neuroangiostrongyliasis in the United States^17^^,^^38^^,^^68^: orange circles, *A. cantonensis* in definitive (rats) and intermediate (snails and slugs) hosts; yellow circles, *A. cantonensis* in nonhuman accidental hosts (see text for details); red circles, numbers of human cases by state and year. AL = Alabama, FL = Florida, HI = Hawaii, LA = Louisiana, MS = Mississippi, OK = Oklahoma, TN = Tennessee, TX = Texas. The parasite is widespread in Florida. In Hawaii (orange and red), there have been human cases on all six largest islands (Table 1), although *A. cantonensis* has not been detected on the island of Lanai.^65^ The record in Oklahoma was a rat (*Sigmodon hispidus*), and one record in Louisiana was also a rat (*Neotoma* sp.), but it is unclear whether either of these species was a definitive or an accidental host.^69^^,^^70^

In the United States, *A. cantonensis* is broadly distributed throughout the Hawaiian Islands,^65^ where the great majority of human cases reported in the country have been contracted since the disease was first identified (Figure 3, Table 1). The parasite is also widely present across Florida, where it has been reported in nonhuman primates, an armadillo, and, in a statewide survey, in roughly 1 in 5 rats and 1 in 50 snails,^66^^,^^75^ yet surprisingly no human cases have been reported there. It is also present in Alabama, Louisiana, Oklahoma, Texas, and possibly Mississippi, based largely on reports in accidental hosts (Figure 3) including nonhuman primates, a horse, armadillos, and an opossum,^66^^,^^67^ with only a handful of autochthonous human cases reported in Texas, Louisiana, Alabama, and one as far north as Tennessee (Figure 3). A small number of additional infections in the continental United States have been detected in travelers returning from endemic parts of the world, including Hawaii (Figure 1).

**Table 1:** Cases of neuroangiostrongyliasis in Hawaii, 1959 to 2021, by island

Year	Island						
	Kauai	Oahu	Maui	Lanai	Hawaii	Unknown	Total
1959–1976	2	15	1	0	1	16	35
1977–1988	1	2	0	0	1	0	4
1989–2000	0	2	0	0	0	0	2
2001–2004	0	12	3	1	4	0	20
2005	0	0	0	0	7	0	7
2006	0	0	0	0	1	0	1
2007	0	0	0	0	2	0	2
2008	0	0	1	0	7	0	8
2009	0	0	0	0	6	0	6
2010	0	1	1	0	7	0	9
2011	0	0	0	0	7	0	7
2012	0	0	0	0	1	0	1
2013	0	0	0	0	3	0	3
2014	0	0	1	0	5	0	6
2015	1	0	0	0	6	0	7
2016	1	0	0	0	11	0	12
2017	0	1	8	0	13	0	22
2018	0	1	0	0	10	0	11
2019	1	0	0	0	8	0	9
2020	0	2	1	0	2	0	5
2021	0	1	1	0	3	0	5
Total	6	37	17	1	105	16	182

Data from Cowie^71^ with additional records from Kuberski and Wallace,^72^ Koo et al.,^73^ Hughes et al.,^74^ the Hawaii Department of Health (https://health.hawaii.gov/docd/resources/reports/summary-of-reported-cases-of-notifiable-diseases/), and Dr. Sarah Kemble, Hawaii Department of Health (personal communication, April 2022).

Despite the fact that *A. cantonensis* is the primary cause of eosinophilic meningitis worldwide, the total number of human infections recorded in the medical literature continues to be surprisingly low, currently ∼ 3,000,^8^ although including additional gray-literature records suggests the number is at least 7,000 (S. Lv, personal communication). However, many more cases have certainly gone unreported, either because symptoms were mild and short-lived and the infected person did not visit a doctor, or because the disease was misdiagnosed. Both of these possibilities are supported by a pilot seroepidemiological study conducted in Hawaii in 2015,^76^ in which 22% of 435 donated human blood specimens tested positive for antibodies to *A. cantonensis* when screened by crude-antigen ELISA and validated by a highly sensitive and specific 31-kDa dot-blot test originally developed in Thailand.

A lack of required reporting in endemic regions is another reason why global estimates of NAS may be woefully inaccurate. In Hawaii, NAS has been a reportable disease since 2007,^77^ and the state Department of Health maintains records of reported cases, with 1 to 22 cases reported per year since 2005 (Table 1). Prior to 2007, cases were identified and reported in the literature based on epidemiological surveys and case series reviews. Elsewhere, records are sketchy at best.

In addition to humans and wildlife, certain domestic and zoo animals can become infected as accidental hosts.^66^ Dogs are of particular interest because they are closely associated with humans and exhibit highly characteristic clinical signs suggesting NAS.^78^ As such, dogs, especially inquisitive, undiscerning puppies (just like some infants and toddlers) that eat or mouth an infected snail, could be considered sentinels for human NAS.^79^

## AN EMERGING BUT NEGLECTED DISEASE

The range of *A. cantonensis*, an invasive species that has not yet reached every favorable locale, continues to expand. Many nonendemic parts of the world are susceptible; for instance, there are no records from most of Africa (Figure 1) and Europe is now threatened.^14^ Several studies of its potential range under climate warming suggest that *A. cantonensis* will extend or shift its range farther from the equator,^69^^,^^80^^,^^81^ including in the continental United States, where the parasite is likely to advance northward over time. In the Hawaiian Islands, its range will expand to include higher areas that are currently too cool for *A. cantonensis*.^65^

## CONCLUSION

Both the medical community and the general public need to become more aware of neuroangiostrongyliasis—a rare but potentially fatal illness. Currently, most medical practitioners in nonendemic regions (and even in Hawaii, to some extent) possess little knowledge of rat lungworm disease. Increased awareness among the medical community will reduce the likelihood of sick people being misdiagnosed in primary care clinics and emergency rooms or simply given analgesics and anxiolytics, or of infected individuals waiting weeks to months before receiving an accurate diagnosis.^68^^,^^82^ In addition, increased awareness leading to prompt diagnosis and treatment will produce more favorable outcomes.^46^^,^^47^ As the parasite spreads, both travelers and residents of newly endemic regions will encounter it more frequently. Therefore, what may be most important of all is effective messaging that does not provoke fear, but allows at-risk residents and visitors to take appropriate precautions to avoid infection in the first place.
